# Research on the Relationship Between Vestibular Migraine With/Without Cognitive Impairment and Brainstem Auditory Evoked Potential

**DOI:** 10.3389/fneur.2020.00159

**Published:** 2020-03-20

**Authors:** Lei Zhang, Qi-hui Chen, Jing-han Lin, Chang Zhou, Yong-hui Pan

**Affiliations:** ^1^Second Affiliated Hospital of Harbin Medical University, Harbin, China; ^2^First Affiliated Hospital of Harbin Medical University, Harbin, China; ^3^HeiLongJiang Red Cross SenGong General Hospital, Harbin, China

**Keywords:** vestibular migraine, migraine, brainstem auditory evoked potential, cognitive impairment, brainstem dysfunction

## Abstract

**Background:** Vestibular migraine (VM) is the most common cause of spontaneous vertigo with no specific physical and laboratory examinations, and is an under-recognized entity with substantial burden for the individual and the society. In this study, by observing the brainstem auditory evoked potential (BAEP) and cognitive function of VM patients, the possible laboratory diagnostic indicators of VM and the influence of disease on cognitive function were discussed.

**Method:** The study included 78 VM patients, 76 migraine patients, and 79 healthy individuals. The age, gender, and other clinical history of the three groups matched. All participants underwent BAEP examinations, in which patients in the migraine group and outpatients of the VM group were in the interictal period, and inpatients in the VM group were examined during episodes, while all patients tested for the Addenbrooke's cognitive examination–revised (ACE-R) scale were in the interictal period. The differences in BAEP and ACE-R scores between the three groups of members and their relationship with the clinical features of VM patients were analyzed.

**Result:** The peak latency of I, III, and V wave in the BAEP of the VM group was longer than that of the migraine group and the control group (*p* < 0.05). The peak latency of V wave in the BAEP of the migraine group was longer than that of the control group (*p* < 0.05). The ACE-R of the VM group scored lower than the migraine group in terms of language fluency and language (*p* < 0.05), and lower than the control group in terms of total score, language fluency, language, and visuospatial (*p* < 0.05); and the ACE-R of the migraine group scored lower than the control group in the total score and visuospatial (*p* < 0.05).

**Conclusion:** Migraine patients have brainstem dysfunction, and VM patients have more severe brainstem dysfunction than migraine patients, suggesting that VM patients have both central nervous system damage and peripheral nerve damage. Migraine patients have cognitive impairment, while cognitive impairment in VM patients is more severe than in migraine patients.

## Introduction

Vestibular migraine (VM) is a clinically common disease with recurrent dizziness/vertigo with nausea and vomiting with or without headache (1). VM is the second most common cause of recurrent vertigo, accounting for about 10% of diseases that can cause dizziness (2, 3).

Brainstem auditory evoked potential (BAEP) is a potential activity change caused by sound stimulation in the brainstem auditory conduction pathway. It is an important neurophysiological indicator reflecting the neurological dysfunction from the cochlea to the brainstem, and also one of the important means to detect brainstem and peripheral nerve function and evaluate audiology (4–6). Wang et al. have confirmed that migraine patients have white matter lesions and cognitive impairment, among which memory, responsiveness, and information recognition and processing ability are all decreased (7). Jonas et al.'s study confirmed the correlation between cognitive function changes and BAEP (8). However, there is still no research on the cognitive function of patients with vestibular migraine. Because of the high incidence of VM and migraine, the research of the medical profession to explore the changes in the function of the brainstem auditory pathway of VM and migraine and its correlation with cognitive function changes is one of the most important subjects.

At present, the literatures at home and abroad have not yet found a study on the relationship between VM and cognitive function changes and BAEP. The purpose of this study is to investigate the changes of BAEP and cognitive function in VM patients, to determine whether the auditory pathway and the brainstem functional state of VM patients have been changed, and to further study whether the pathogenesis of VM is related to brainstem and peripheral nerves. Furthermore, the diagnostic significance of BAEP in the VM with or without cognitive function changes is analyzed to provide theoretical basis for the prevention and clinical treatment of VM, and it is of great significance for the study of epidemiology and clinical diagnostic indicators of VM.

## Materials and Methods

### Patients

Seventy-eight VM patients were from the First Affiliated Hospital of Harbin Medical University. The time was from July 2018 to December 2018. Twenty-one outpatients were in the interictal period and 57 inpatients were in the attack period. There were 11 males and 67 females with an average age of 51.77 ± 11.58 years. All of the patients met the VM ICHD-3 beta diagnostic criteria and were diagnosed by both a clinical otolaryngologist and a clinical neurologist. The migraine group included 76 patients, including 16 males and 60 females, with an average age of 39.20 ± 13.69 years, all of whom met the diagnostic criteria for ICHD-3 beta and were diagnosed by a clinical neurologist. The above patients were enrolled using the following criteria: 18 years of age or older; the number of attacks in half a year no less than twice; vertigo or headache attack in the most recent month; get informed consent from the participants. The exclusion criteria are as follows: patients diagnosed with other types of chronic headache (such as tension headaches); diagnosis of other types of dizziness (e.g., benign paroxysmal positional vertigo [BPPV], Meniere's disease, etc.); patients with hearing impairment; patients with other chronic disease history (such as cerebrovascular disease, tumor, hypothyroidism, hyperthyroidism, diabetes, chronic obstructive emphysema, Parkinson's disease, AIDS, hypertension, heart disease, blood disease, kidney failure, liver functional failure, Cushing's syndrome, anxiety, and depression, etc.); and taking cortisol medications within 1 week. At the same time, 79 healthy people without vertigo were recruited as controls, including 22 males and 57 females with an average age of 49.00 ± 14.97 years. All subjects signed informed consent and were approved by the Ethics Committee of the First Hospital of Harbin Medical University.

### Method

All participants were required to provide a detailed medical history to a trained neurologist (whether there was a history of vertigo, accompanying symptoms, predisposing factors, other related diseases, etc.) and basic vital signs (temperature, blood pressure, heart rate measurement). All participants underwent BAEP examinations, in which patients in the migraine group were in the interictal period, outpatients of the VM group were in the interictal period, and inpatients in the VM group were examined during episodes. The participants were quiet and supine. The BAEP detection application evoked potential meter model is the Danish KEYPOINT 8000 model. The test method is as follows: the reference electrode is located on the ipsilateral mastoid of the test subject, the recording electrode is located in the middle of the head (Cz), the ground is located in the forehead (Fpz), the impedance is <5 K ohm, the stimulus is sent to the ear through an earphone (air conduction), and the test ear is, respectively, stimulated by 120 and 100 dBnHL click sound. For acoustic stimulation, the side ear is given a 40-dBnHL white noise mask to reduce interference from other factors. The sound stimuli are superimposed 1,000 times, and each ear is repeated at least two times and the single ear is separately stimulated. Sources of BAEP waveforms are as follows: I wave represents the electrical activity of the extracranial segment of the auditory nerve; II wave represents the potential change of the cochlear nucleus and the intracranial segment of the auditory nerve; III wave represents the electrical activity of the olive nucleus and the cochlear nucleus on the brainstem; IV wave represents the change in medial lemniscus; V waves represent changes in the potential of the inferior colliculus.

**Figure 1:**
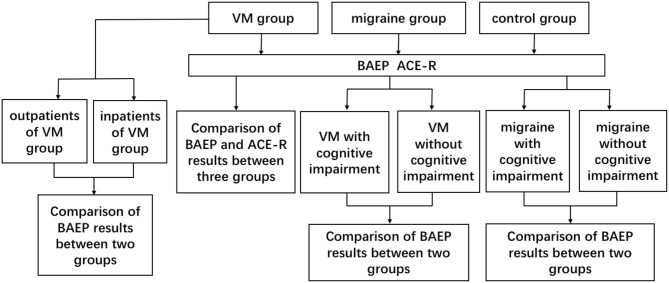
Flow diagram of experiment.

All patients participating in cognitive function tests were evaluated by an experienced neurologist using the ACE-R (the Addenbrooke's cognitive examination–revised). All patients were tested for the ACE-R during the interparoxysms. The ACE-R is a comprehensive cognitive assessment scale designed and revised by the University of Cambridge. The scale provides a detailed assessment of the following five aspects: orientation or attention (18), memory (26), language fluency (14), language (26), and visuospatial (16); the total score is 100, and the higher the score, the better the cognitive function. The ACE-R has a very high sensitivity and specificity at 83 in the diagnosis of cognitive impairment. In view of the differences in cultural background, scholars from different countries also modified the corresponding version of ACE-R after translating and adjusting the culture (9, 10). Further evaluation was conducted by researchers who were trained and unaware of the results of the test. See Figure 1 for the specific test process.

### Observation Index

BAEP observation indicators: 1. The peak latency of I, III, V waves (PL), waveform differentiation good rate. 2. I–III/III–V peak-to-peak latency ratio. 3. The difference between each wave PL ear. BAEP abnormality judgment criteria: 1. The main wave I, III, V disappear or the repeatability is poor; 2. III–V/I–III IPL>1; 3. The average value of each wave PL is increased by three standard deviations compared with the control group; 4. The difference between the PL ears of each wave is >0.4 ms. Observe the score of each sub-item of the ACE-R scale.

### Statistical Method

The main researcher used Microsoft Excel 2016® to record and store the data. A descriptive analysis of the data that considered absolute and relative frequencies, central tendency measures (average and median), and dispersion measures (standard deviation, minimum and maximum values) was performed.

For quantitative variables, the standard distribution was verified, and Student's *t*-test was used to compare the groups. The equality of variance (standard deviation square) was not assumed when the homogeneity of a certain variable could not be confirmed. For the association analyses between independent qualitative variables and outcome measures, the chi-square test was used. For statistical significance, a descriptive level of 5% (*p* < 0.05) was considered. Data were analyzed with the Statistical Package for the Social Sciences (SPSS).

## Results

### Differences in BAEP Between VM Group, Migraine Group, and Control Group

**Figure 2:**
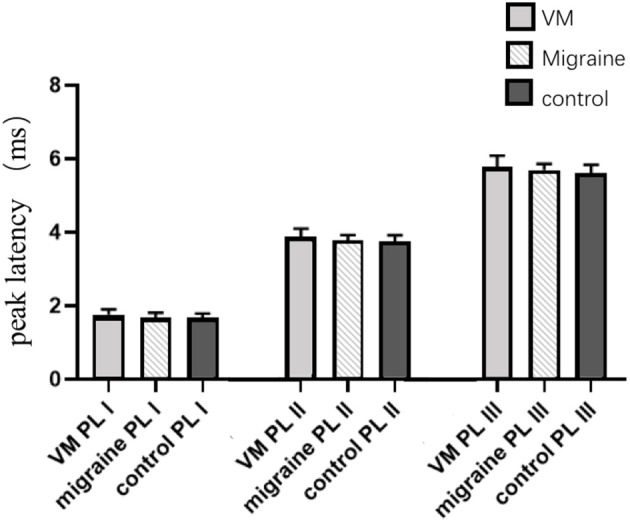
Comparison of BAEP results between the three groups.

**Figure 3:**
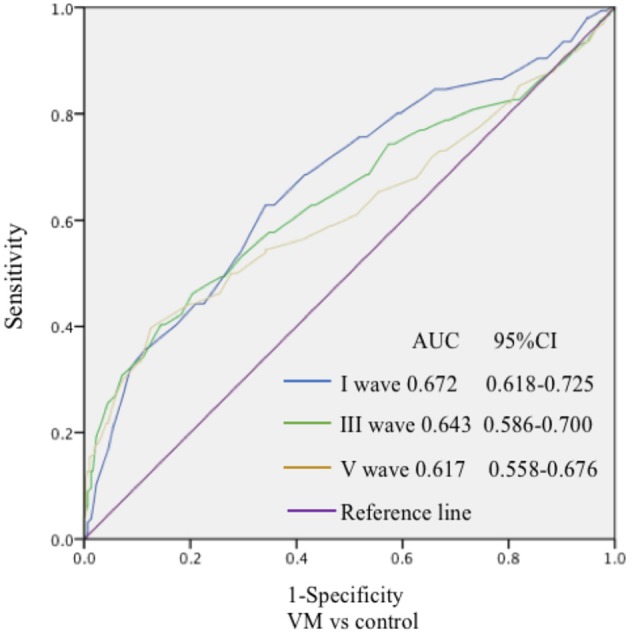
The specificity and sensitivity of the ROC curve to the diagnostic value of BAEP. The ROC curve was drawn to evaluate the specificity and sensitivity of the diagnostic value of BAEP. The highest point in the upper left corner was close to 1, indicating good specificity and sensitivity of the model.

**Figure 4:**
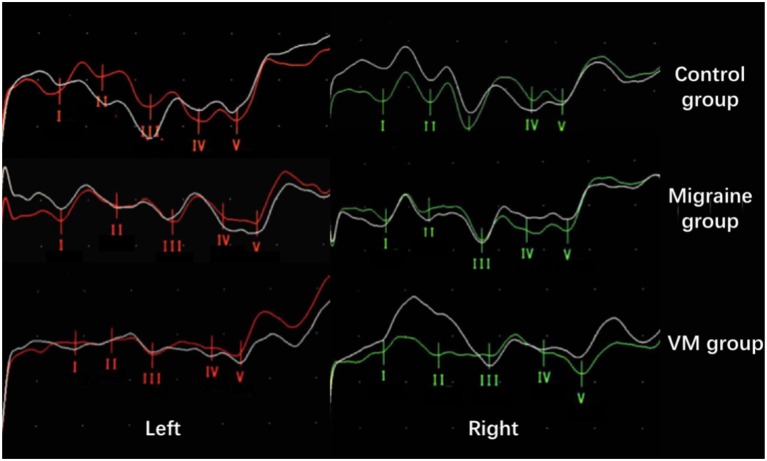
Comparison of BAEP amplitude results between the three groups (from top to bottom: control group; migraine group; VM group). Compared with the control group, the BAEP results of the migraine group showed decreased IV and V wave amplitude. Compared with the migraine group, the BAEP results of the VM group showed decreased amplitude of I and II wave, while the amplitude of I–V wave was decreased in the VM group compared with the control group.

In this study, 78 patients with VM had a duration of dizziness >5 min, <72 h, of which 5 min to 1 h accounted for 35.8%, 1 h to 1 day accounted for 43.5%, and >1 day accounted for 20.7%. All patients underwent routine neurological examination. Among the 78 patients in the VM group, 47 patients had different degrees of abnormalities in BAEP, and the abnormal rate was 60.25%, including seven males and 40 females. Among the 76 patients in the migraine group, 24 patients had different degrees of abnormalities in BAEP, and the abnormal rate was 31.58%, including six males and 18 females. Among the 79 patients in the control group, eight patients had different degrees of abnormalities in BAEP, and the abnormal rate was 10.13%, including two males and six females (Table 1). There was no statistically significant difference in the relative ratio of left and right ears in the VM group and the migraine group of BAEP (*p* > 0.05) (Table 2). The mean BAEP values for the three groups' subjects are presented in Figure 2. In the VM group, the average peak latency of I wave in the BAEP was 1.76 ± 0.14 ms, the average peak latency of III wave was 3.87 ± 0.23 ms, and the average peak latency of V wave was 5.78 ± 0.31 ms. In the migraine group, the average peak latency of I wave in BAEP was 1.69 ± 0.13 ms, the average peak latency of III wave was 3.77 ± 0.15 ms, and the average peak latency of V wave was 5.70 ± 0.17 ms. In the control group, the average peak latency of I wave in the BAEP was 1.68 ± 0.11 ms, the average peak latency of III wave was 3.75 ± 0.17 ms, and the average peak latency of V wave was 5.61 ± 0.22 ms. The migraine group data were compared with the control group data; the peak latency of V wave was prolonged (*p* < 0.05). The peak latency of I, III, and V wave of the VM group was significantly longer than that of the other two groups (*p* < 0.05) (Table 3). Meanwhile, the roc curve was used to evaluate its diagnostic value (Figure 3). However, comparing the BAEP results of the outpatients of the VM group between the inpatients of the VM group, the peak latency of I wave was prolonged, which was statistically significant (Table 4). In addition, compared with the control group, the BAEP results of the migraine group showed decreased IV and V wave amplitude. Compared with the migraine group, the BAEP results of the VM group showed decreased amplitude of I and II wave, while the amplitude of I–V wave was decreased in the VM group compared with the control group (Figure 4). We compared the BAEP results of the three groups of patients according to different genders again. In male patients, the peak latency of I wave of the VM group was longer than that of the control group, and the peak latency of I and V wave of the migraine group was longer than that of the control group. Among female patients, the peak latency of I, III, and V wave of the VM group was longer than that of the migraine group; the peak latency of III and V wave was longer than that of the control group; and the peak latency of V wave of the migraine group was longer than that of the control group (Table 5).

**Table 1:** Comparison of baseline data between the three groups.

**Characteristics**	**VM group** **(*n* = 78)**	**Migraine group** **(*n* = 76)**	**Control group** **(*n* = 79)**
Age, mean ± SD (years)	51.77 ± 11.58	45.20 ± 13.69	49.00 ± 14.97
Women/men	11/67	16/60	22/57
BAEP abnormalities rate	60.25%	31.58%	10.13%
Medical history (years)	11.03 ± 12.11	9.74 ± 12.13	–
Duration (hour)	18.09 ± 25.41	16.2 ± 20.37	–
Attack number of times	8.65 ± 11.08	9.66 ± 18.95	–

**Table 2:** Comparison of BAEP results between left and right ears in the three groups (±s, ms).

	**VM group** **(*****n*** **=** **78)**		**Migraine group** **(*****n*** **=** **76)**		**Control group** **(*****n*** **=** **79)**		***Pa* value**	***Pb* value**	***Pc* value**
	**Left**	**Right**	**Left**	**Right**	**Left**	**Right**			
PL I	1.76 ± 0.14	1.77 ± 0.15	1.67 ± 0.12	1.70 ± 0.13	1.67 ± 0.11	1.69 ± 0.11	0.951	0.899	0.655
PL III	3.86 ± 0.22	3.88 ± 0.24	3.77 ± 0.14	3.77 ± 0.16	3.74 ± 0.18	3.75 ± 0.16	0.668	0.111	0.337
PL V	5.75 ± 0.28	5.80 ± 0.34	5.71 ± 0.14	5.69 ± 0.19	5.61 ± 0.23	5.62 ± 0.22	0.209	0.195	0.890

*Pa, comparison between left and right ears in the VM group; Pb, comparison between left and right ears in the migraine group; Pc, comparison between left and right ears in the control group*.

**Table 3:** Comparison of BAEP results between the three groups (±s, ms).

	**VM group** **(*n* = 78)**	**Migraine group** **(*n* = 76)**	**Control group** **(*n* = 79)**	***Pa* value**	***Pb* value**	***Pc* value**
PL I	1.76 ± 0.14	1.68 ± 0.13	1.68 ± 0.11	0.045	0.207	0.001
PL III	3.87 ± 0.23	3.77 ± 0.15	3.75 ± 0.17	0.000	0.269	0.000
PL V	5.78 ± 0.31	5.70 ± 0.17	5.61 ± 0.22	0.000	0.000	0.001

*Pa, comparison between the VM group and the migraine group; Pb, comparison between the migraine group and the control group; Pc, comparison between the VM group and the control group*.

**Table 4:** Comparison of BAEP results between VM patients in outpatient and ward (±s, ms).

	**Outpatients of VM group** **(*n* = 21)**	**Inpatients of VM group** **(*n* = 57)**	***P-*value**
PL I	1.77 ± 0.11	1.76 ± 0.16	0.003
PL III	3.90 ± 0.24	3.85 ± 0.23	0.944
PL V	5.79 ± 0.28	5.77 ± 0.32	0.314

**Table 5:** Comparison of BAEP results between different gender in the three groups (±s, ms).

	**VM group**		**Migraine group**		**Control group**		***Pa*** **value**	***Pb*** **value**	***Pc*** **value**	***Pd*** **value**	***Pe*** **value**	***Pf*** **value**
	**Male** **(*n* = 11)**	**Female** **(*n* = 67)**	**Male** **(*n* = 16)**	**Female** **(*n* = 60)**	**Male** **(*n* = 22)**	**Female** **(*n* = 57)**						
PL I	1.84 ± 0.13	1.75 ± 0.14	1.69 ± 0.13	1.68 ± 0.13	1.68 ± 0.09	1.68 ± 0.12	0.331	0.159	0.000	0.098	0.033	0.812
PL III	4.05 ± 0.20	3.84 ± 0.22	3.79 ± 0.15	3.76 ± 0.15	3.80 ± 0.14	3.73 ± 0.17	0.365	0.000	0.167	0.013	0.598	0.210
PL V	6.08 ± 0.26	5.73 ± 0.29	5.75 ± 0.17	5.68 ± 0.16	5.72 ± 0.21	5.57 ± 0.21	0.069	0.000	0.472	0.008	0.110	0.000

*Pa, comparison between male patients in the VM group and the migraine group; Pb, comparison between female patients in the VM group and the migraine group; Pc, comparison between male patients in the VM group and the control group; Pd, comparison between female patients in the VM group and the control group; Pe, comparison between male patients in the migraine group and the control group; Pf , comparison between female patients in the migraine group and the control group*.

### Differences in ACE-R Between VM Group, Migraine Group, and Control Group

**Figure 5:**
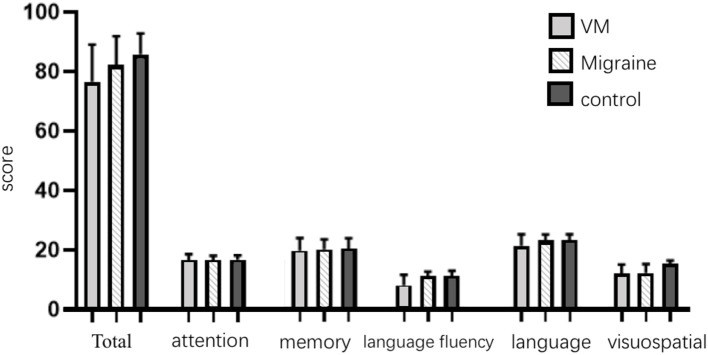
Comparison of ACE-R results between the three groups.

The mean ACE-R score values for the three groups' subjects are presented in Figure 5. There were 60 patients with cognitive impairment (total score <83) and 18 with normal cognitive function (total score ≥ 83) in the VM group. The ACE-R score of VM patients had the language fluency score of 7.86 ± 3.54 and the language score of 21.23 ± 3.80, which were lower than the language fluency score of 10.90 ± 1.60 and the language score of 22.95 ± 1.97 of the migraine group (*p* < 0.05). There were 38 patients with cognitive impairment (total score <83) and 38 with normal cognitive function (total score ≥ 83) in the migraine group. The ACE-R score of migraine patients had a total score of 82.29 ± 9.56 and a visual spatial score of 12.03 ± 3.01, which were lower than the total score of 85.80 ± 7.00 and the visual spatial score of 15.19 ± 1.00 of the control group (*p* < 0.05). The scores of ACE-R in VM patients were lower than those in controls in total score, language fluency, language, and visuospatial (*p* < 0.05) (Table 6). Finally, the VM group was divided into the VM cognitive function impairment group (total score <83) and the VM cognitive function normal group (total score ≥ 83), and the comparison showed that there was no significant difference between the peak latency of I, III and V wave periods of the two groups (*p* > 0.05). The migraine group was divided into the group with migraine cognitive impairment (total score <83) and the group with migraine normal cognitive function (total score ≥ 83), and it was found that there were no significant differences in the peak latency of I, III, and V between the two groups (*p* > 0.05) (Tables 7, 8).

**Table 6:** Comparison of ACE-R results between the three groups.

	**VM group** **(*n* = 78)**	**Migraine group** **(*n* = 76)**	**Control group** **(*n* = 79)**	***Pa* value**	***Pb* value**	***Pc* value**
Total	76.60 ± 12.48	82.29 ± 9.56	85.80 ± 7.00	0.633	0.002	0.010
Attention	16.31 ± 2.13	16.46 ± 1.53	16.54 ± 1.47	0.386	0.702	0.251
Memory	19.50 ± 4.31	19.96 ± 3.30	20.17 ± 3.51	0.351	0.439	0.722
Language fluency	7.86 ± 3.54	10.90 ± 1.60	10.92 ± 1.81	0.000	0.449	0.000
Language	21.23 ± 3.80	22.95 ± 1.97	22.98 ± 2.09	0.000	0.689	0.000
Visuospatial	11.69 ± 3.22	12.03 ± 3.01	15.19 ± 1.00	0.573	0.000	0.000

*Pa, comparison between the VM group and the migraine group; Pb, comparison between the migraine group and the control group; Pc, comparison between the VM group and the control group*.

**Table 7:** Comparison of BAEP between cognitive impairment group and normal cognitive function group in VM patients (±s, ms).

	**VM cognitive impairment group** **(*n* = 60)**	**VM normal cognitive function group** **(*n* = 18)**	***P*-value**
PL I	1.75 ± 0.13	1.77 ± 0.15	0.195
PL III	3.88 ± 0.18	3.86 ± 0.25	0.057
PL V	5.80 ± 0.24	5.77 ± 0.33	0.054

**Table 8:** Comparison of BAEP between cognitive impairment group and normal cognitive function group in migraine patients (±s, ms).

	**Migraine cognitive impairment group** **(*n* = 38)**	**Migraine normal cognitive function group** **(*n* = 38)**	***P*-value**
PL I	1.69 ± 0.12	1.68 ± 0.13	0.668
PL III	3.77 ± 0.16	3.77 ± 0.15	0.161
PL V	5.69 ± 0.16	5.70 ± 0.18	0.158

## Discussion

VM is a very common disease that causes recurrent vertigo, but its lack of clinical understanding leads to a serious underestimation of its prevalence (11). The diagnostic criteria for VM were proposed in the International Classification of Headache Disorders (ICHD-3 beta) in 2013, which provides a strong basis for the diagnosis of VM (12, 13). BAEP is widely used to assess the functional impairment of the brainstem auditory pathway in the central nervous system and auditory system diseases (14). The pathophysiological mechanisms of VM are not fully understood, so studying the relationship between VM and BAEP can help provide more certainty for the diagnostic assessment and clinical management of these patients in everyday clinical practice.

In this study, compared with the migraine group and the control group, the peak latency of I, III, and V wave in the VM group was significantly prolonged (*p* < 0.05). Compared with the control group, the peak latency of V wave in the migraine group was prolonged (*p* < 0.05). Those with an abnormal single ear may have less lesions and do not involve the contralateral conduction pathway, while those with abnormal ears may have electrophysiological changes to the contralateral side due to the presence of large lesions in the conduction pathway. The peak latency of I wave in the outpatients of the VM group was longer than the inpatients of the VM group indicating that the hearing impairment of VM patients was fluctuating and correlated with the onset of symptoms. VM patients in the outpatient department are in the convalescence period, and their auditory nerve function damage is more serious than that in the inpatient department. We speculate that there is no clear correlation between auditory nerve function damage and the onset of vertigo symptoms in VM patients, and auditory nerve function damage may fluctuate. Of course, this result may also be related to the error caused by the unequal number of patients in the two groups. The peak latency of V wave changes is not obvious, indicating that the brainstem function damage in VM patients may be long term. However, due to the mismatch in the number of patients in the two groups, there may be a result error, so the difference in BAEP between the attack and interictal patients should be further studied.

VM is more common in women, which may be related to familial disease-causing genes and hormone levels (1). We compared the differences in BAEP among members of the three groups of different genders. In male patients, the VM group and the migraine group had a longer latency of the peak latency of I wave than the control group. In female patients, the peak latency of I, III, and V wave in the VM group was longer than that in the migraine group, and the peak latency of III and V wave was longer than that of the control group; the peak latency of V wave in the migraine group was longer than that in the control group. Lopez et al. demonstrated that the latency of BAEP is influenced by age and gender, with the peak latency of III and V wave in females significantly shorter than in males (15). Our data showed that the changes in the peak latency of III and V wave of BAEP in the female patients of the VM group were more significant than those in the male patients. This may indicate that women are more vulnerable to damage to the function of the auditory nerve to the brainstem nerve, leading to a higher incidence of VM in women. However, the causes of functional impairment of auditory nerve to brainstem nerve in women need to be further studied.

According to reports, BAEP reflects the extent of auditory pathways and brainstem ischemia and is extremely sensitive to synaptic dysfunction (16). In this study, the BAEP results of the VM group were compared with those of the migraine group, which confirmed that decreased auditory nerve function may be one of the causes of VM. There is a large overlap between the migraine pathway and the vestibular pathway. The trigeminal vasculature has been shown to affect the blood supply to the inner ear in animal experiments (17). The cochlear region has a terminal capillary bed. The high metabolic requirements of the inner ear and the hereditary nature of the cochlea make it impossible to form collateral vessels, which is highly sensitive to a very small reduction in blood supply (5, 18). The parasympathetic nerve affects the trigeminal vascular reflex, leading to cerebral vasospasm and reduced blood flow, which in turn triggers a sterile inflammatory response and the release of vasoactive substances (e.g., histamine, 5-HT, and plasma kinin) (5, 19), resulting in ischemia of the inner ear and inducing vertigo. Drugs (such as triptans, ergotrine, and CGRP antagonists) act on the trigeminal innervation area to attenuate VM specific peripheral triggers.

The V wave in BAEP represents the electrical activity in the upper part of the pons or in the lower middle part of the brain, which originates from the inferior colliculus. There are multiple serotonin pathways in the dorsal raphe nucleus and the inferior colliculus, and serotonin from the dorsal raphe nucleus is introduced into the vestibular nucleus (20, 21). Serotonin is an important regulator of auditory processing and usually coexists with gamma-aminobutyric acid (GABA) in the inferior colliculus (4). Intravenous serotonin in mice has been shown to increase the extravasation of vestibular nerve proteins (22). Sand et al. (4) also confirmed a negative correlation between serotonin levels and the IV–V wave amplitude during the onset of migraine patients, and this association was more pronounced in VM. Therefore, serotonin changes in the inferior colliculus may be one of the reasons for the prolongation of the peak latency of V wave and may be one of the potential causes of vestibular dysfunction in VM (23). The tightly connected network between the blue spot and the dorsal raphe nucleus may be a target for calcitonin gene-related peptide antagonists and triptans in VM. Changes in BAEP can reflect changes in midbrain neurotransmitters and/or hypoperfusion (24). Bhargava et al. (25) observed that high-dose kappa choline (1–5 mg/kg) significantly increased the peak latency and amplitude of BAEP III and IV waves in rats, while low-dose cholinergic activity inhibited BAEP, demonstrating the effect of cholinergic activity on BAEP. Reserpine induced prolongation of III wave and V wave peak in rats, suggesting that norepinephrine and serotonin have an effect on BAEP (26). The inferior colliculus receives serotoninergic input from the dorsal raphe nucleus. Serotonin from the nucleus can also regulate the upper olive nucleus and the cochlear nucleus, which has an effect on the BAEP III wave (24). In a longitudinal study by Sand et al. (4), the intensity dependence of BAEP in migraine may not be a passive response to brainstem dysfunction. There were no changes in BAEP before the onset of migraine, and the peak latency of I–V wave and the peak latency of III–V wave increased after the onset of headache; the changes in BAEP seemed to reflect the slight effect of migraine symptoms on the serotonin brain stem pathway (27, 28). In this study, patients in the migraine group had a longer V wave latency period than those in the control group. Compared with the migraine group, the peak latency of I, III, and V wave in the VM group were prolonged, but the V wave changes were still within the normal range, indicating that the inner ear and cochlear core function changes were involved in the occurrence of VM. Meanwhile, brainstem function was decreased in migraine patients, and brainstem dysfunction was more serious in VM patients than in migraine patients. Due to the high sensitivity of BAEP, slight functional abnormalities in the brainstem and inferior colliculus have been discovered. Thus, BAEP shows a mild population abnormality in VM patients and has implications for the pathogenesis of VM.

Long-term recurrent attacks on the VM can lead to cognitive impairment. Wang et al. (7) conducted a controlled trial of VM patients and healthy people, which concluded that the cognitive dysfunction of VM patients was more serious. At the same time, the imaging examination of VM patients found that the incidence of white matter lesions was higher than that of normal people. In this experiment, we calculate the ACE-R, which has its unique advantages in non-memory assessment such as executive function, visuospatial, attention, and directionality. Higher specificity and sensitivity were found for disease assessment of cognitive impairment manifested by attention or directivity impairment and executive function impairment. The ACE-R is highly sensitive, specific, and powerful in assessing cognitive impairment and early detection of cognitive impairment and related diseases. In this study, the cognitive function of VM patients was significantly reduced in terms of language and language fluency compared with migraine patients, while the cognitive function of migraine patients was reduced in visuospatial compared with controls. However, it has not been found to be associated with BAEP. Repeated episodes of migraine, repeated painful stimulation of patients, gradually appearing cognitive impairment, and emotional changes such as anxiety and depression, difficulty in concentration and other psychological problems, in the long run, have a serious negative impact on patients' quality of life. In the imaging study of cognitive impairment in VM patients, brain function was found to be concentrated in the parietal lobe and the hippocampus. Brandt et al. (29) reported that 17% of patients with bilateral vestibular dysfunction found atrophy in the hippocampus. Stimulation of the vestibular nucleus can lead to more obvious electrophysiological changes in the hippocampus. Patients with VM also have unilateral vestibular dysfunction (29, 30). At the same time, cognitive impairment of VM patients is mainly manifested in language fluency, language, and spatial function. The visual areas in the prefrontal and temporal lobes (mainly including the temporal pole, frontal lobe, fusiform gyrus, and inferior temporal gyrus) and the medial occipital lobe significantly inhibit the visual information in the vestibular cortex pathway, which is similar to the fear of sound and light in migraine patients. Damage to the vestibular function leads to impairment of cognitive function, and cognitive impairment also acts on the vestibular organs, affecting each other, causing a vicious circle (31). In the past, in clinical practice, the damage of cognitive function was often neglected due to the headache and vertigo symptoms of VM patients. This study confirmed the cognitive function impairment of VM patients, which can provide scientific reference for early clinical treatment and prevention of VM and reduce the risk of cognitive function impairment of VM patients. However, this study did not find a correlation between cognitive impairment and BAEP changes in VM patients and migraine patients.

Our research highlights the following points: (1) The peak latency of I wave in the VM group were prolonged in VM patients, although the left and right contrasts are not statistically significant. We believe that these results are caused by the impairment of the auditory nerve function, which may have certain diagnostic significance for the pathophysiological changes of VM. Meanwhile, there was no statistical difference between the migraine group and the control group in the peak latency of I wave. Therefore, we believed that inner ear impairment and synaptic dysfunction of VM were different from migraine, which was one of the causes of vertigo. (2) Compared with the control group, the peak latency of V wave was prolonged in migraine patients. At the same time, the peak latency of V wave in VM patients was longer than that in migraine patients, but it was still within the normal range, indicating that VM patients may have brainstem dysfunction but it was mild. We hypothesized that norepinephrine and serotonin produced by the blue-spot and dorsal raphe nucleus of the brainstem may influence the signaling of the inferior thalamus and the function of the central nervous system, thus inducing vertigo symptoms. (3) Cognitive impairment in migraine patients mainly involves visuospatial aspects, while VM patients also have language fluency and language impairment. (4) There is no correlation between cognitive impairment and BAEP changes in VM patients and migraine patients.

Although the results of this study are important, there are still some limitations: (1) the relatively small number of patients; (2) some VM patients were examined by BAEP and ACE-R scale during the recovery period of dizziness, which may affect the experimental results; and (3) the temporal relationship between the appearance of BAEP changes and the onset of VM symptoms due to the cross-sectional design of this study. BAEP was not repeated during the VM symptom onset. The relationship between cognitive impairment and the onset time of VM needs further study.

## Conclusion

VM is a benign recurrent vertigo with migraine and dizziness. Its recurrent episode has a serious impact on the quality of life of patients. It is a disease in which peripheral and central mechanisms coexist, and there are functional changes from the inner ear to the brainstem.

BAEP can detect different functions of nerve nuclei and nerve conduction pathways, and can reflect minor damage. Although it is not of great diagnostic significance, it can hint the pathogenesis of VM to some extent.

VM patients have cognitive impairment and need to follow up regularly to track the degree of change. Meanwhile, appropriate physical therapy and health education guidance can be provided to improve the prognosis of VM patients, provide the basis for treatment, and point out the direction.

## Data Availability Statement

The datasets generated for this study are available on request to the corresponding author.

## Ethics Statement

The studies involving human participants were reviewed and approved by Ethics Committee of the first affiliated hospital of Harbin Medical University. The patients/participants provided their written informed consent to participate in this study.

## Author Contributions

YP: study design. YP and LZ: participant recruitment. LZ: data collection, data analysis, and manuscript preparation. QC, JL, and CZ: manuscript preparation during revision.

### Conflict of Interest

The authors declare that the research was conducted in the absence of any commercial or financial relationships that could be construed as a potential conflict of interest.

## References

[1] Furman JM Marcus DA Balaban CD. Vestibular migraine: clinical aspects and pathophysiology. Lancet Neurol. (2013) 12:706–15. 10.1016/S1474-4422(13)70107-8 23769597

[2] Kang WS Lee SH Yang CJ Ahn JH Chung JW Park HJ. Vestibular function tests for vestibular migraine: clinical implication of video head impulse and caloric tests. Front Neurol. (2016) 7:166. 10.3389/fneur.2016.00166 27746761 PMC5044462

[3] Radtke A Neuhauser H von Brevern M Hottenrott T Lempert T. Vestibular migraine–validity of clinical diagnostic criteria. Cephalalgia. (2011) 31:906–13. 10.1177/0333102411405228 21508087

[4] Sand T Zhitniy N Stovner LJ. Brainstem auditory-evoked potential habituation and intensity-dependence related to serotonin metabolism in migraine: a longitudinal study. Clin Neurophysiol. (2008) 119:1190–200. 10.1016/j.clinph.2008.01.007 18316245

[5] Hamed SA Youssef AH Elattar AM. Assessment of cochlear and auditory pathways in patients with migraine. Am J Otolaryngol. (2012) 33:385–94. 10.1016/j.amjoto.2011.10.008 22133970

[6] Vander Werff KR Rieger B. Brainstem evoked potential indices of subcortical auditory processing after mild traumatic brain injury. Ear Hear. (2017) 38:200–14. 10.1097/AUD.0000000000000411 28319479 PMC5482771

[7] Wang N Huang HL Zhou H Yu CY. Cognitive impairment and quality of life in patients with migraine-associated vertigo. Eur Rev Med Pharmacol Sci. (2016) 20:4913–7. 27981543

[8] Jonas BK Wilson WJ Sebastian W Increasing cognitive interference modulates the amplitude of the auditory brainstem response. J Am Acad Audiol. (2018) 29:512–9. 10.3766/jaaa.17003 29863465

[9] Wong L Chan C Leung J Yung CY Wu KK Cheung SY. A validation study of the Chinese-Cantonese Addenbrooke's Cognitive Examination Revised (C-ACER). Neuropsychiatr Dis Treat. (2013) 9:731–7. 10.2147/NDT.S45477 23785235 PMC3682856

[10] Fang R Wang G Huang Y Zhuang JP Tang HD Wang Y. Validation of the Chinese Version of Addenbrooke's Cognitive Examination-Revised for screening mild Alzheimer's disease and mild cognitive impairment. Dementia Geriatr Cogn Disord. (2014) 37:223–31. 10.1159/000353541 24193223

[11] Carvalho GF Vianna-Bell FH Florencio LL Pinheiro CF Dach F Bigal ME. Presence of vestibular symptoms and related disability in migraine with and without aura and chronic migraine. Cephalalgia. (2019) 39:29–37. 10.1177/0333102418769948 29635938

[12] Headache Classification Committee of the International Headache Society (IHS) The International Classification of Headache Disorders, 3rd edition (beta version). Cephalalgia. (2013) 33:629–808. 10.1177/0333102413485658 23771276

[13] Ferster APOC Priesol AJ Isildak H The clinical manifestations of vestibular migraine: a review. Auris Nasus Larynx. (2017) 44:249–52. 10.1016/j.anl.2017.01.014 28285826

[14] Thirumala PD Carnovale G Habeych ME Crammond DJ Balzer JR. Diagnostic accuracy of brainstem auditory evoked potentials during microvascular decompression. Neurology. (2014) 83:1747–52. 10.1212/WNL.0000000000000961 25298303

[15] López Escámez JA Salguero G Salinero J Age and sex differences in latencies of waves I, III and V in auditory brainstem response of normal hearing subjects. Acta Oto Rhino Laryngol Belg. (1999) 53:109–15. 10427363

[16] Santos MARM Munhoz SL Silva MALPS. High click stimulus repetition rate in the auditory evoked potentials in multiple sclerosis patients with normal MRI. Rev Laryngol Otol Rhinol. (2004) 125:151−5. 15605430

[17] Furman JM Balaban CD. Vestibular migraine. Ann NY Acad Sci. (2015) 1343:90–6. 10.1111/nyas.12645 25728541

[18] He JW Gong Q Wang XF Xiao Z. High stimulus rate brainstem auditory evoked potential in benign paroxysmal positional vertigo. Eur Arch Otorhinolaryngol. (2014) 272:2095–100. 10.1007/s00405-014-3172-6 25005432

[19] Yollu U Uluduz DU Yilmaz M Yener HM Akil F Kuzu B. Vestibular migraine screening in a migraine-diagnosed patient population, and assessment of vestibulocochlear function. Clin Otolaryngol. (2017) 42 :225–33. 10.1111/coa.12699 27385658

[20] Jeong SH Oh SY Kim HJ Koo JW Kim JS. Vestibular dysfunction in migraine: effects of associated vertigo and motion sickness. J Neurol. (2010) 257:905–12. 10.1007/s00415-009-5435-5 20041331

[21] Holland PR. Modulation of trigeminovascular processing: novel insights into primary headache disorders. Cephalalgia. (2010) 29(s3):1–6. 10.1177/03331024090290S302 20017748

[22] Koo JW Balaban CD. Serotonin-induced plasma extravasation in the murine inner ear: possible mechanism of migraine-associated inner ear dysfunction. Cephalalgia. (2010) 26:1310–9. 10.1111/j.1468-2982.2006.01208.x 17059438

[23] Brodsky JR Mejico LJ Giraud A Woods CI III. Impairment of habituation of the auditory brain stem response in migrainous vertigo. Ann Otol Rhinol Laryngol. (2013) 122:308–15. 10.1177/000348941312200504 23815047

[24] Hall IC Hurley LM. The serotonin releaser fenfluramine alters the auditory responses of inferior colliculus neurons. Hear Res. (2007) 228:82–94. 10.1016/j.heares.2007.01.023 17339086 PMC1950579

[25] Bhargava V Salamy KA Mckean CM. Effect of cholinergic drugs on the brainstem auditory evoked responses (far-field) in rats. Neuroscience. (1978) 3:821–6. 10.1016/0306-4522(78)90034-9 714253

[26] Morales-Martinez J González-Piña JR Alfaro-Rodríguez A Brainstem auditory response in the reserpinized rat. Proc West Pharmacol Soc. (2002) 45:68−70. 12434532

[27] Li M Wang C. Brainstem auditory-evoked potential and migraine: is there an association? Med Princ Pract. (2015) 24:296. 10.1159/000369932 25573236 PMC5588215

[28] Sürmeli M Sürmeli R Deveci I Önder S Yalçin AD Oysu Ç. Correlation between cVEMP and ABR for the evaluation of vestibular migraine. J Int Adv Otol. (2016) 12 :326–31. 10.5152/iao.2016.2338 27879227

[29] Brandt T Dieterich M. Vestibular contribution to three-dimensional dynamic (allocentric) and two-dimensional static (egocentric) spatial memory. J Neurol. (2016) 263:1015–6. 10.1007/s00415-016-8067-6 26946497

[30] Popp P Wulff M Finke K Rühl M Brandt T Dieterich M. Cognitive deficits in patients with a chronic vestibular failure. J Neurol. (2017) 264:1–10. 10.1007/s00415-016-8386-7 28074268

[31] Harun A Oh ES Bigelow RT Studenski S Agrawal Y. Vestibular impairment in dementia. Otol Neurotol. (2016) 37:1137–42. 10.1097/MAO.0000000000001157 27466890 PMC5392114

